# The Many Lives of [Ru(bpy)_3_]^2+^: A Historical Perspective

**DOI:** 10.1021/acs.inorgchem.5c03471

**Published:** 2025-11-18

**Authors:** Giorgio Scattolini, Andrea Rosichini, Nidhi Kaul

**Affiliations:** Department of Chemistry − Ångström Laboratory, 8097 Uppsala University, Box 523, 751 20 Uppsala, Sweden

## Abstract

[Ru­(bpy)_3_]^2+^, tris­(bipyridine)­ruthenium­(II), is a popular transition metal complex whose favorable photophysical properties have afforded it a central place in inorganic photochemistry and various related fields. In this perspective, in contrast to the large number of extant technical reviews, we instead note critical developments from a historical context. Of particular note are relatively lesser-known investigations in the field of analytical chemistry that predate the complex’s rise to prominence as a photosensitizer. Recent studies that revisit the complex’s own fundamental photophysics are also highlighted. Thus, in addition to serving as a proverbial almanac for the complex’s rich history, this condensed perspective portends yet more fruitful lives for research into [Ru­(bpy)_3_]^2+^, despite the many already lived.

## Graphical abstract

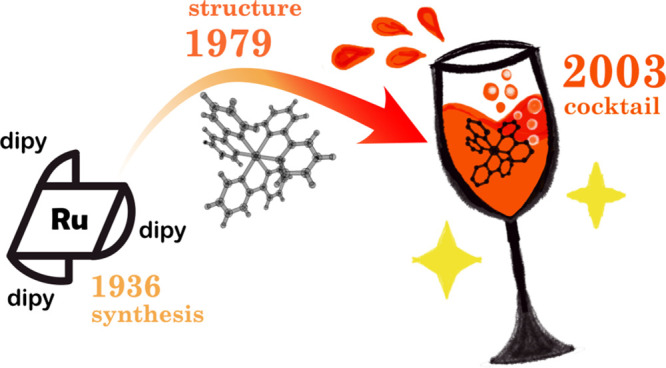

## Introduction

Cleverly christened as the “fruitfly of photophysics” by Professor Oliver Wenger at the University of Basel,^1^ tris­(bipyridine)­ruthenium­(II) (Figure 1a), [Ru­(bpy)_3_]^2+^, has assumed center stage in the development of inorganic photophysics. The fact cannot be overstated: one need only look at the collated Web of Science output seen in Figure 1b (averaging well over a hundred papers a year since the 1990s) to appreciate the magnitude of the impact. This does not include close cousins inspired by the archetypal motif; one might then suspect the number grows significantly. In this brief historical journey, we map landmark publications involving [Ru­(bpy)_3_]^2+^ (Figure 2). These include the first report of its synthesis by Burstall,^2^ when it was second-fiddle to congener [Fe­(bpy)_3_]^2+^, leading up to the many debates surrounding its fundamental photophysics,^3–6^ and plethora of applications.^7–9^ The fact that a single coordination complex has captured the imagination of generations of chemists can be traced to its unique optical properties. Indeed, with excited state reduction potentials of −0.86 and 0.84 V (vs NHE) in water, appreciable absorptivity in the visible (ϵ_454 nm_ = 14,600 M^–1^ cm^–1^), a lifetime of 620 ns, and an emission quantum yield of 0.042 in deaerated water,^4,10^ [Ru­(bpy)_3_]^2+^ presents the benchmark against which newly discovered photosensitizers are evaluated even today.

**Figure 1:**
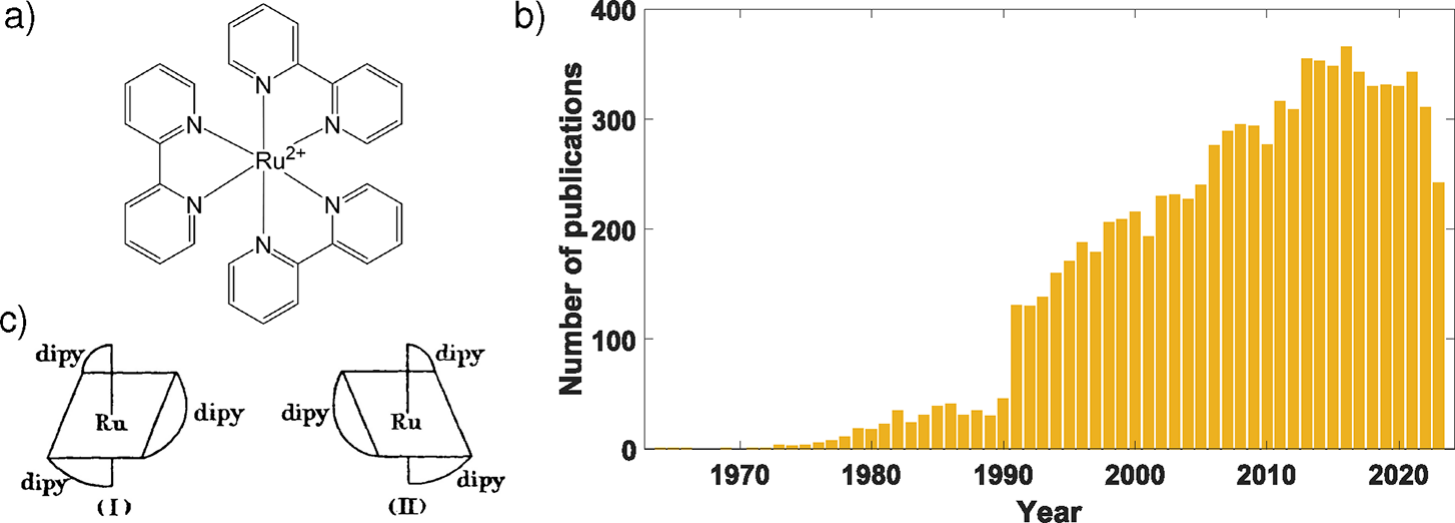
(a) Chemical structure of [Ru­(bpy)_3_]^2+^. (b) Web of Science output for “Tris­(bipyridine)­ruthenium” or “Tris­(dipyridine)­ruthenium” or “Ru­(bpy)­3” or “ruthenium trisbipyridine” or “Ru­(dipy)­3” or “Rubpy3” or “Rudipy3” or “Tris­(2,2′-bipyridine) Ruthenium”, i.e., various monikers for [Ru­(bpy)_3_]^2+^ over the past several decades. (c) Structures of the two enantiomers of [Ru­(bpy)_3_]^2+^ reported by Burstall in 1936. Reproduced with permission from ref 2. Copyright 1936 Royal Society of Chemistry.

**Figure 2:**
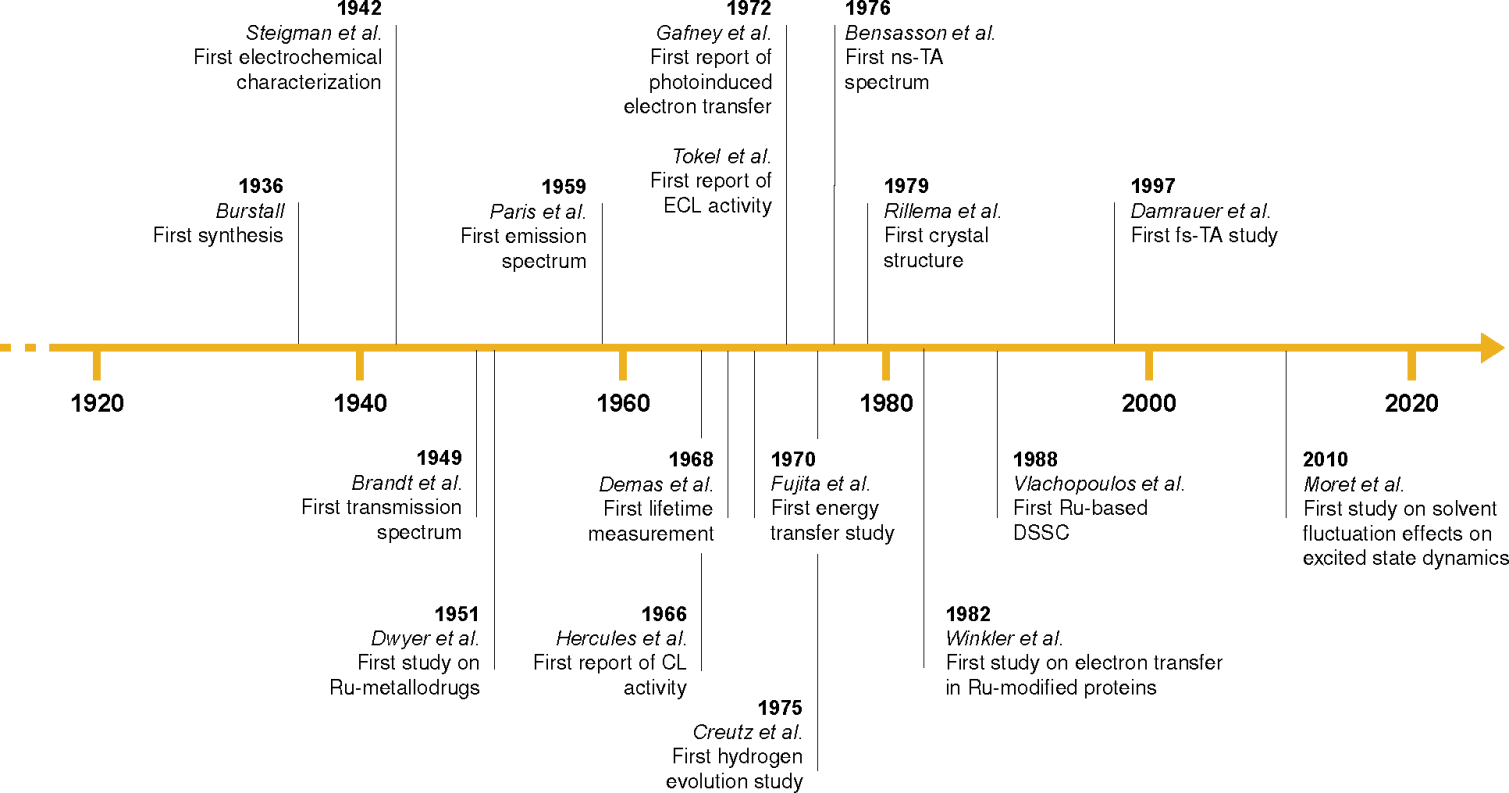
Timeline of the key publications in the history of [Ru­(bpy)_3_]^2+^ (CL: chemiluminescence; ECL: electrochemiluminescence; TA: transient absorption; DSSC: dye-sensitized solar cell).

Remarkably, as we shall find through the course of this interlude, a history of [Ru­(bpy)_3_]^2+^ when put in a broader context is not just one of transition metal photophysics. Rather, it subsumes landmark developments in coordination chemistry, spectroscopy, analytical chemistry, and photochemistry more generally. Accordingly, in the following sections, we trace the cation’s trajectory from being an overlooked colorimeter to being reborn as one of the most prominent photosensitizers known today, making every effort to capture both the breadth and depth of impact. After completing our condensed survey, we present a concluding outlook: what lives remain for [Ru­(bpy)_3_]^2+^ to live, if any?

## Inorganic Chemistry

The history of the bipyridine (bpy) ligand – without which the central subject of this treatise would not exist – has already been documented by Constable et al. in an exhaustive review.^11^ Here, we merely note that Fritz Blau in 1888 reported the first synthesis of bipyridine together with the first observation of the complexation of Fe by it.^12^ He would also later report the synthesis of another popular polypyridyl, ortho-phenanthroline (phen).^13^ Afterward, in 1936, Francis Burstall reported the first synthesis of [Ru­(bpy)_3_]^2+^ (the structures of the two enantiomers drawn by Burstall are reported in Figure 1C).^2^ Mechanistic details of the synthesis were not elucidated until 1955 by Miller, Brandt and Puke;^14^ Brandt was in fact the one to report some of the first spectroscopic investigations on [Ru­(bpy)_3_]^2+^ as well.

At the time he synthesized [Ru­(bpy)_3_]^2+^, Burstall was working at the Chemical Research Laboratory (UK),^15^ under the supervision of Gilbert Thomas Morgan, one of the more prominent inorganic chemists of his time,^16^ who, among other things, first implemented the term “chelate”.^17^ Prior to this, Burstall and Morgan had already reported the first synthesis of terpyridine (terpy),^18^ its derivatives – including [Ru­(terpy)_2_]^2+^ ^19,20^ – and investigated other polypyridyl complexes of silver,^21^ nickel,^22^ and platinum.^23,24^ The paper by Burstall on [Ru­(bpy)_3_]^2+^ focused on comparing the Ru compound to the Fe and Ni derivatives reported by Blau^12,13^ and the studies on their optical activity by Werner^25^ and Burstall himself.^22^ He already noted that [Ru­(bpy)_3_]^2+^ is significantly more stable compared to the first-row analogues and that it does not undergo racemization, not even at high temperatures. He ends the paper by noting that “Tris-2:2′-dipyridylruthenous salts dye silk and wool in orange-yellow shades.”. Overall, this first synthetic report can be seen in the context of research on the structural properties of coordination compounds and the reactivity of polypyridines. A field that was approaching its end, since Werner already got his Nobel prize in 1913 “[for] his work on the linkage of atoms in molecules [···] especially in inorganic chemistry”.^26^ The famed photophysical life of [Ru­(bpy)_3_]^2+^ would not surface until spectrometers became more commonplace: as we shall see in the next section, however, the familiar pale orange color of the complex would instead afford it a place in the realm of analytical chemistry.

## Analytical Chemistry

Colorimetric determination methods of metals have been around since the time of the Roman Empire. Pliny reported in 60 CE the determination of iron with a mixture based on vinegar.^27^ However, at the time, it was mostly based on qualitative approaches, relying on natural light^28^ or quantitative approaches, but relying on the judgment of the operator. An extensive review of these methods was compiled in 1921 by Snell.^29^ Despite these titration methods having been reported since the 18th century, research was still ongoing in the first half of the 20th century.^30,31^ In particular, the field of redox titration was still under development: its applications were limited due to the lack of reversible indicators. This scenario changed at the beginning of the 20th century, however, as reversible indicators started to be discovered.^31,32^

Among the first were studies by Hammet and Walden^33^ in the 1930s, on iron complexes as redox indicators. They found that the phen complex was more suitable than the bpy due to its higher stability in acidic conditions.^34^ This discovery was readily recognized for its relevance and prompted further investigations for the use of transition-metal complexes (TMCs) as redox indicators.^35^

This is the context in which the second article on [Ru­(bpy)_3_]^2+^ appeared. In 1942, Steigman et al. studied the potential use of [Ru­(bpy)_3_]^2+^ as a redox indicator and noted that Walden suggested studying this system.^36^ In this study, first observations were made regarding the great stability of the oxidized form and the reversibility of the redox reaction. These were desirable properties for colorometric applications; the reduction potential of 1.33 V vs NHE for the Ru^3+^/Ru^2+^ couple was also reported for the first time. As for the study by Burstall, it was inspired by previous results on the use of iron complexes, especially phenanthrolines, as indicators.

In 1946, Dwyer and Nyholm reported the instability constants of both phen and bpy iron complexes, noting the interest in these compounds for analytical applications.^37^ Together with Humpoletz, they reported the first synthesis of [Ru­(phen)_3_]^2+^ and investigated its potential applications as a redox indicator, inspired by the previous study by Steigman et al. on [Ru­(bpy)_3_]^2+^. A follow-up paper by Dwyer investigated the effect of ligand substitution on the reduction potentials of [Ru­(phen)_3_]^2+^, demonstrating the possibility of fine-tuning these parameters by engineering the ligand of the complexes.^38^ These publications were part of a series of studies from the Australian community of coordination chemistry, aimed at systematic investigation of metal-polypyridyls.^39^ In general, at the time, Ru was chasing Fe: it was only used in niche applications; alloying agent for Pt and Pd in electrical contact and jewelry, and for the fabrication of tips for fountain pens and long-lasting phonograph needles. The costs proved prohibitive, with Ru costing between 790,000 and 200,000 times more than Fe between 1941 and 1959 in the United States,^40,41^ so much so that grants could be acknowledged specifically for its purchase.^2,42^

Optical properties of Ru-polypyridines did show great promise, however. The rotatory power of [Ru­(phen)_3_]^2+^ was also measured by Backhouse and Dwyer in 1949, and they found a minimal tendency toward racemization,^43^ much as Burstall had noted for [Ru­(bpy)_3_]^2+^. Furthermore, it was shown that oxidation does not induce racemization of [Ru­(bpy)_3_]^2+^.^44^ This was in contrast to the Fe-polypyridines, which were far more prone to racemization. Earnest investigations into the optical properties from a photophysical perspective would not begin until decades later, as we detail in a subsequent section.

For the extant period, [Ru­(bpy)_3_]^2+^ nevertheless entrenched itself as an important redox titer, even if second fiddle to Fe, and was also central in the swiftly emergent branch of analytical chemistry for the spectrophotometric determination of metals via formation of complexes. In light of rising industrial interest in ruthenium, a series of methods for its spectrophotometric determination appeared in the literature in the 1940s and 1950s.^45–47^ This was enabled by the birth of the earliest spectrometers between the 1920s and 1930s, and their eventual commercialization in the 1940s, making them more accessible and affordable.^28,48–50^

It was in this context that Brandt first reported the transmission spectrum of [Ru­(bpy)_3_]^2+^ in 1949 (Figure 3a). The study concerned the application of bpy and phen complexes as redox indicators and for spectrophotometric analysis of metals, especially iron.^51,53^ It is worthwhile to note that in the preceding decade, Yamasaki had reported the first absorption spectra of many polypyridine complexes, although not [Ru­(bpy)_3_]^2+^, making it one of the first reports on the spectroscopic characterization of TMCs.^54^

**Figure 3:**
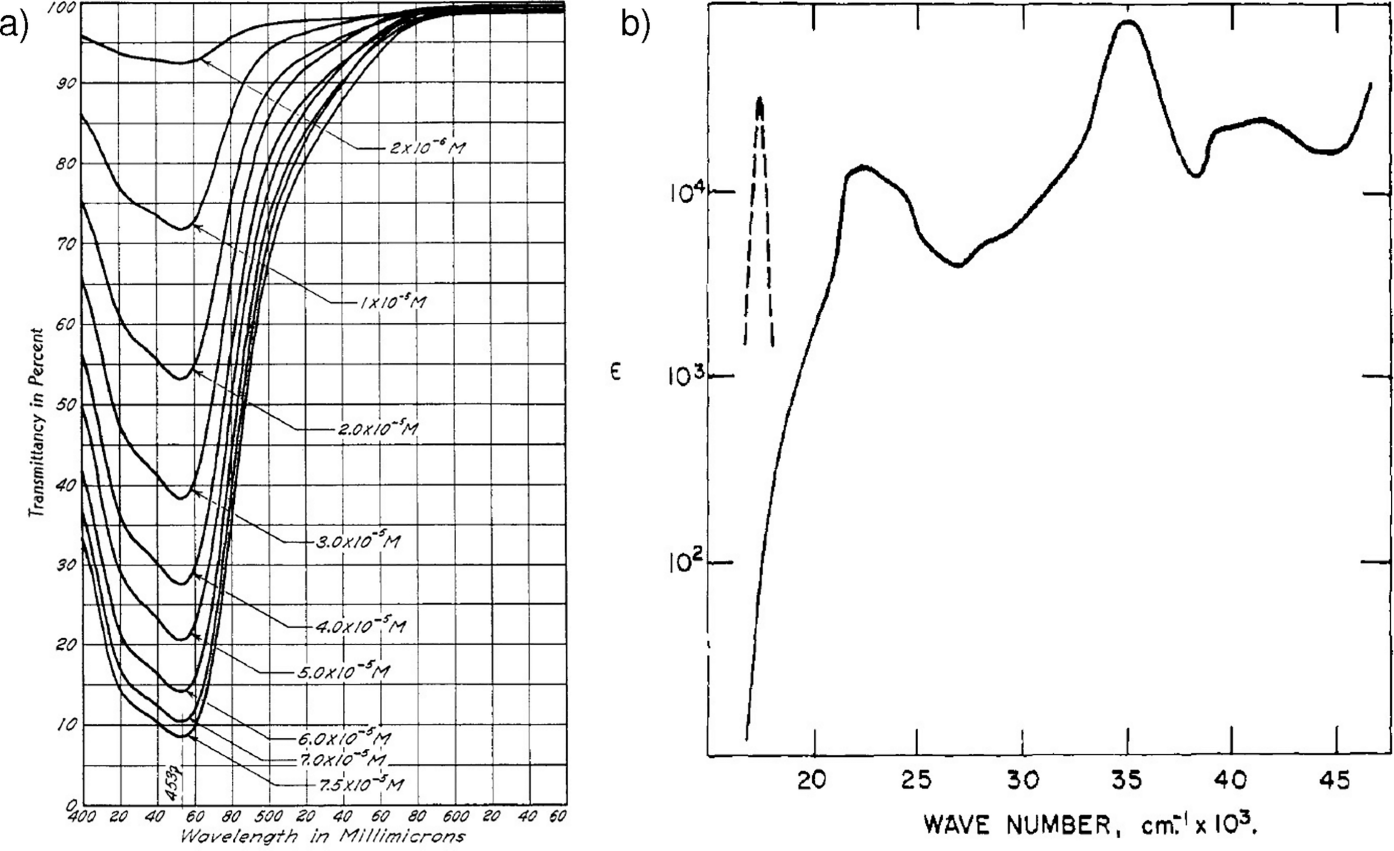
(a) First transmission spectrum of [Ru­(bpy)_3_]^2+^ reported by Brandt et al. in 1949. Reproduced from ref 51. Copyright 1949 American Chemical Society. (b) First emission spectrum (dashed line) reported by Paris et al. in 1959. Reproduced from ref 52. Copyright 1959 American Chemical Society.

Another photophysical property used for the determination of analytes was, of course, luminescence spectrometry. The use of emission as a parameter for analysis has been known since before luminescence was scientifically investigated. Already in the 19th century, many analytical protocols were developed based on emission.^55,56^ One of the first reports of TMC-based emission was made by Randall in 1944, who reported the emission of Pt­(bpy)­Cl_2_.^57^

However, the turning point was the introduction of the first fluorimeter between the end of the 1950s and the beginning of the 1960s.^50,55,58–60^ In 1959, Paris and Brandt were also the first to report the emission spectra of [Ru­(bpy)_3_]^2+^ (Figure 3b).^52^ They correctly assigned the luminescence to a charge transfer transition but arbitrarily labeled it as fluorescence. Veening and Brandt had actually already observed [Ru­(bpy)_3_]^2+^ emission in a previous study (but published later in 1960) on fluorometric determination of Ru by complexation with bpy and phen.^61^ However, since the best results were obtained with the Me-phen ligand, the spectrum of [Ru­(bpy)_3_]^2+^ was not shown or discussed.

We conclude this section with a summarizing statement from Paris from his dissertation in 1960, “The investigation of the area of charge transfer electronic transitions in metal chelates was undertaken in an effort to increase the understanding and exploit the analytical utility of their absorption and luminescence spectra.”.^62^ As we shall see in the next section, these initial discoveries would give way to motivations of a more photophysical and eventually photochemical character.

## Photophysics and Photochemistry

Charge transfer states and the associated transitions were studied and discussed since the 1920s^63,64^ but only became relevant since the 1940s when a theory to model them started to be developed.^65–67^ However, the experimental work was focused mostly on materials,^68,69^ while molecules were playing a minor role and experiments were limited to absorption spectroscopy.^70–73^ In his doctoral thesis, Paris states: “[···] most of the work has come from solid state physicists rather than the synthetic organic chemists”.^62^ Accordingly, the previously mentioned 1959 report of [Ru­(bpy)_3_]^2+^’s luminescence^52^ was not only a milestone in the history of the molecule, but that of molecular photophysics generally. Indeed, it was one of the first explicit assignments of emission from a charge-transfer transition in a molecule.

This triggered an entire suite of studies in the 1960s and 1970s, which sought to delineate the complex’s excited-state photophysical behavior. This rise from hibernation would prove transformative, marking the beginning of [Ru­(bpy)_3_]^2+^’s most prolific life. A chronological compilation of these fundamental findings was made by Kalyanasundaram in an exhaustive, seminal review in 1982, where he aptly notes, “For the characterisation of the excited state, there is hardly any other transition metal complex or organic molecule that has received more careful scrutiny and attention than the complex [Ru­(bpy)_3_]^2+^.”.^4^ Spanning over 80 pages and 350 references, the review speaks to a watershed moment in the investigation of [Ru­(bpy)_3_]^2+^ (and derivatives, including [Ru­(phen)_3_]^2+^). The interested reader is directed there for details and also to another foundational compilation made by Juris et al. in 1988.^10^ In the following paragraphs, we present a suitably redacted version, marking key developments and subjects, which are also illustrated in Figure 2.

At first, even the initial assignment of the emission’s origin as π*-d CT by Paris and Brandt was disputed. Porter and Schläfer suggested a d*-d ligand field phosphorescence,^69^ while Crosby, Perkins, and Klaasen made a d*-d fluorescence assignment.^74^ Of the many studies that contributed to the 15-year controversy, a systematic investigation - mostly led by Crosby and co-workers, that also reported the first estimation of the emission lifetime^75^ – could ultimately establish the triplet charge-transfer nature of the emitting state.^4^ In tandem, reports on reactivity also began to emerge: the first example of energy transfer from [Ru­(bpy)_3_]^2+^ to [Cr­(ox)_3_]^3–^ was shown by Fujita and Kobayashi in 1970 in a mixed crystal at 77 K.^76^ The following year, in 1971, Demas and Adamson reported the quenching of [Ru­(bpy)_3_]^2+^ by [Pt­(Cl)_4_]^2–^ as the first example of room temperature energy transfer between two complexes.^77^ In this paper, ligand exchange in [Pt­(Cl)_4_]^2–^ was observed as a result of energy transfer, hinting at the possibility of driving reactions using [Ru­(bpy)_3_]^2+^. In 1972, Gafney and Adamson published the first study on photoinduced electron transfer from [Ru­(bpy)_3_]^2+^ to a series of Co­(III) complexes.^78,79^ At the time, energy transfer to the Co­(III) species in some of the investigated systems could not be ruled out, but it could be shown that [Ru­(bpy)_3_]^2+^ was getting oxidized.

The aforementioned series of papers on photoinduced electron transfer involving [Ru­(bpy)_3_]^2+^ have been considered as one of the key moments in the history of this molecule and, more generally, for the field of inorganic photochemistry, since such reactivity had been rarely observed before in TMCs.^80,81^ Demas (who previously worked with Crosby) and Adamson further explored the possibility of using [Ru­(bpy)_3_]^2+^ to trigger chemical reactions, via energy or electron transfer, in a series of oxalate complexes.^82^ Since then, [Ru­(bpy)_3_]^2+^ began to be used as photosensitizer in mechanistic studies.^83,84^ Notably, Natarajan et al. explicitly mentioned the choice of [Ru­(bpy)_3_]^2+^ given that its “emission spectroscopy and utility as a sensitizer have been well documented”.^84^ The same year as the photoinduced electron transfer studies with [Ru­(bpy)_3_]^2+^ were reported, Fujishima and Honda’s landmark paper on the first demonstration of photoelectrochemical water splitting with TiO_2_ was published.^85^ With the backdrop of the extant socioeconomic climate, characterized by the 1973 oil crisis, conditions thus became fertile for further research on [Ru­(bpy)_3_]^2+^ as a photosensitizer, this time for semiconductors. This could be seen as part of a renewed impetus on research in artificial photosynthesis^86,87^ (since Ciamician already proposed such a vision at the beginning of the century^88^), which also resulted in the determination of [Ru­(bpy)_3_]^2+^’s crystal structure (Figure 4).^89^ Ultimately, this would lead to the sensitization of semiconductors with dyes to improve the efficiency of photoelectrochemical devices, bringing about the dye-sensitized solar cells (DSSCs) we know today. We note critical developments in the relevant subsection.

**Figure 4:**
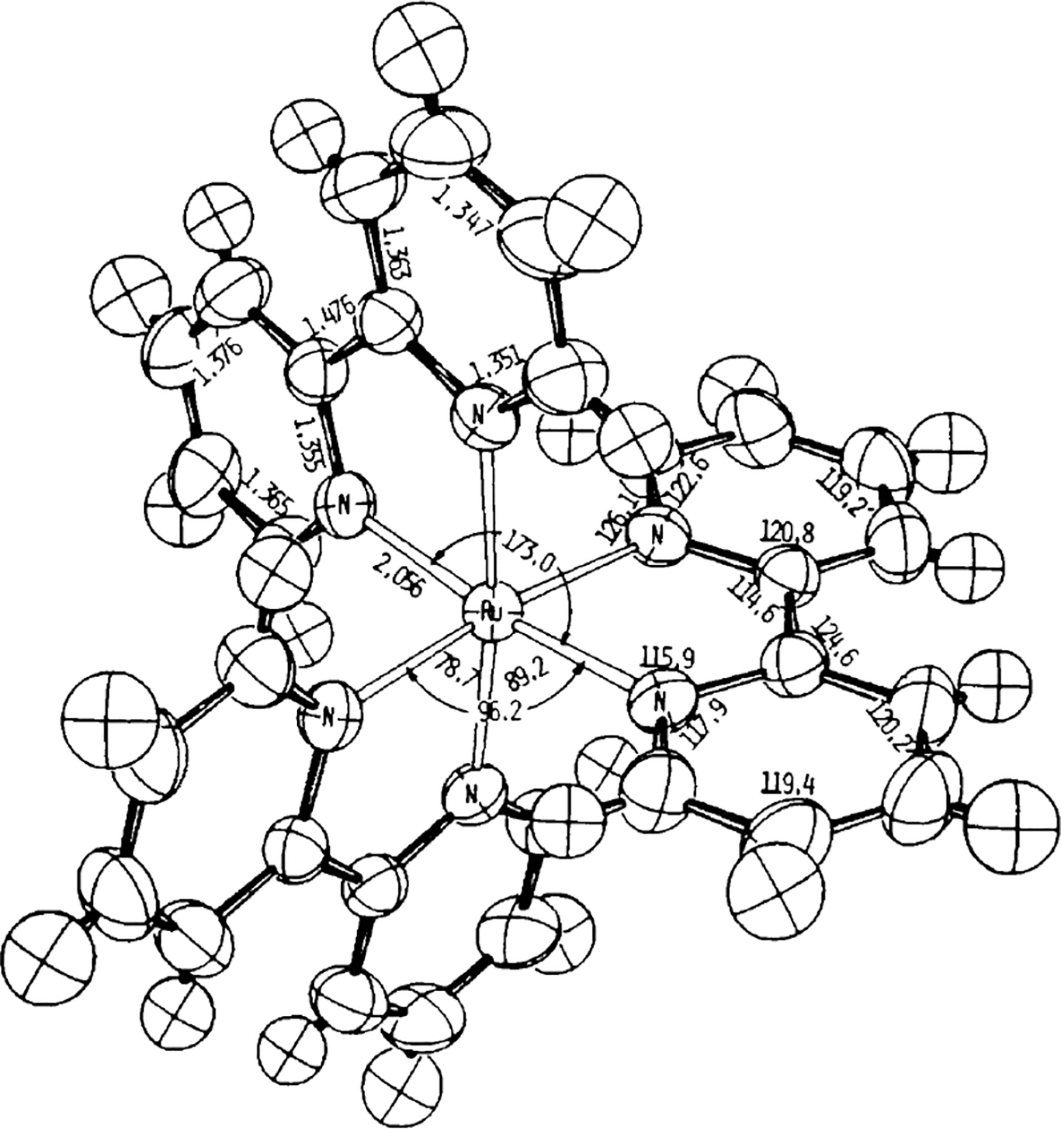
First crystal structure of [Ru­(bpy)_3_]^2+^ reported by Rillema et al. in 1979. Reproduced with permission from ref 89. Copyright 1979 Royal Society of Chemistry.

Meanwhile, avenues for further photochemical and mechanistic investigations were far from exhausted. In the early 1970s, many papers appeared in the literature investigating the quenching mechanism of [Ru­(bpy)_3_]^2+^. These studies were mostly focusing on quenching via energy transfer, with TMCs as quenchers.^83,90–96^ A seminal study was published in 1974 by Bock, Meyer, and Whitten. They reported spectroscopic evidence of photoinduced electron transfer from [Ru­(bpy)_3_]^2+^ to methyl viologen (the birth of the only true love story), trans-1,2-bis­(*N*-methyl-4-pyridyl)­ethylene, and Fe^2+^.^97^ This study represented a turning point as it clearly demonstrated single-electron transfer involving organic molecules, at a time when electron transfer quenching was not fully established yet.^81^ In parallel to the aforementioned study, Navon and Sutin also reported evidence for electron transfer quenching of [Ru­(bpy)_3_]^2+^ by Co and Ru complexes.^92^ These mechanistic investigations would eventually give way to the creation of molecular “wires” with [Ru­(bpy)_3_]^2+^, bridging units, and other complexes to precisely control the rate of electron and energy transfer.^98,99^ Synthesis and characterization of multinuclear species containing [Ru­(bpy)_3_]^2+^ was also pioneered by the school of photochemistry at the University of Bologna,^80^ and with the advent of supramolecular chemistry, large dendritic structures incorporating [Ru­(bpy)_3_]^2+^ ^100,101^ were reported by the late 90s, for both light-harvesting and sensing applications – work which has continued in the past decade as well.^102^

A first attempt to measure picosecond processes of [Ru­(bpy)_3_]^2+^ was made by Kirk et al. in 1976.^103^ However, the experimental limitations of their apparatus prevented the retrieval of meaningful information. In the same year, Bensasson, Salet and Balzani reported the first nanosecond transient absorption spectrum that further confirmed the triplet metal-to-ligand charge-transfer (^3^MLCT) nature of the emission.^104^

Although the MLCT nature of the emission was firmly established by the late 1970s, questions remained with regard to the fundamental photophysical landscape. Of particular interest was the number of the emissive ‘triplet’ electronic states, their energies, as well nature, i.e., localized or delocalized.^105^ Low-temperature luminescence polarization measurements first reported by Fujita and Kobayashi in 1973 showed a high polarization value of ≈0.23, suggesting localization.^106^ By 1987, Carroll and Brus reported picosecond time-resolved resonance Raman spectroscopy on [Ru­(bpy)_3_]^2+^, unequivocally establishing that the electron was localized on one bpy ligand on long time scales.^107^ Early temperature-dependent lifetime and quantum yield measurements by Hager and Crosby could be fit to a phenomenological model based on multiple emitting MLCT states with an equilibrated Boltzmann population distribution.^108^ Separations of ca. 10 and 50 cm^–1^ could be calculated between the triplet levels.^109^ Gallhuber, Hensler and Yersin reported single-crystal polarized emission measurements in 1985, which showed sharp line emissions in agreement with this earlier work, and a potential fourth state ca. 200 cm^–1^ above the lower energy levels.^110^ The empirical Kober–Meyer model has been developed to explain the data.^111^ Work by Myrick, Blakley and De Armond disputes such a model, however, instead suggesting three spin-states separated by 0.1 cm^–1^, as in a typical aromatic heterocycle.^112^ Excited state electron spin resonance results by Yamauchi, Komada and Hirota , with calculated zero-field splitting of *g* ≈ 0.2 and *D* = 0.1 cm^–1^, were found consistent with such a picture.^113^ The two contrasting views are summarized by Yersin et al.^114^ and De Armond^5^ in two early accounts, and the reader is directed there for details. Further questions remained, however, regarding the origins of the localization (intrinsic or solvent-induced), as well as electron localization/delocalization on short time scales. The latter has remained a contentious issue for decades for this *D*_*3*_ symmetry complex – one which seems unresolved even at the time of writing: half a century of debate!^6^

In the 1980s, laser technology progressed to the point of generating fs laser pulses.^115^ This led to the development of the field of femtochemistry,^116^ for which Zewail was awarded the Nobel Prize in 1999. The first fs-TA study on [Ru­(bpy)_3_]^2+^ was carried out in 1997 by Damrauer, McCusker et al., which was also the first one on a TMC. This could readily confirm the ultrafast nature of the intersystem crossing process from ^1^MLCT to ^3^MLCT,^117^ with the mechanism theoretically suggested only recently, in 2017.^118^ Bhasikuttan, Okada et al. reported the first femtosecond fluorescence upconversion measurement on [Ru­(bpy)_3_]^2+^ in 2002, wherein an intersystem crossing time of 40 ± 15 fs could be estimated.^119^ Four years later, Canizzo, Chergui et al. reported spectral observation of an invariant ^1^MLCT emission and intersystem crossing on sub-30 fs time scales, confirming the first result.^120^ Previous Stark spectroscopy measurements made by Oh and Boxer in 1989 had already shown a significant magnitude of the dipole moment, suggesting a localized ^1^MLCT excited state.^121^ Taken together, these results all but established the localized nature of the excited state on all time scales.

Several follow-up reports^122–125^ investigated the ultrafast charge localization on the ligand(s), with disparate observations. These could be reconciled by a 2015 study from Stark, Kohler, et al. The latter showed interligand electron-hopping in [Ru­(bpy)_3_]^2+^ could take place on multiple time scales, ranging from sub-ps (“hot” interligand electron transfer) to tens of ps, with the rates influenced by the amount of excess vibrational excitation energy.^126^ Details to this point have been compiled by Dongare, Meyer et al. for ready reference.^6^

On the theoretical front, an important study by Moret, Tavernelli et al., employing hybrid DFT and molecular dynamics simulations, was published in 2010. They suggested solvent-induced localization of the electron on one or two bpy ligands, with more prevalence of the latter.^127^ They further noted electron-hopping between the ligands on time scales of ca. half a picosecond in water.^128^ Follow-up theoretical investigations corroborating the claims of the study have appeared, in support of a “two-ligand localization” model.^129,130^ Experimental evidence for these theoretical findings has also emerged, contrasting with the prevailing consensus, and key studies are summarized below.

Notable is the investigation by Stockett and Nielsen, reported in 2015,^131^ examining the intricacies of solvent-induced localization (over two decades after De Armond and Myrick had suggested such experiments^5^). Briefly, the gas-phase photodissociation action spectra of isolated [Ru­(bpy)_3_]^2+^, and when solvated by a single acetonitrile molecule, were found to be identical. A collective solvent effect was thus found to be necessary for localization, with complete delocalization not only in the gas phase, but also with a single acetonitrile present. In a 2019 report, Stark, Rebane et al. employed femtosecond two-photon transient absorption measurements to probe the change in permanent dipole moment in the excited state.^132^ They found theoretical results in agreement with the Moret model and further suggested that the large, nonzero dipole moment observed in the excited state was a consequence of solute–solvent interactions.

The most recent, and arguably one of the most interesting works providing further insight, was published in 2022 by Pelczarski, Stampor et al.^133^ Using electroabsorption (EA) measurements, the MLCT excited state was found to be delocalized and orbitally degenerate in neat films of [Ru­(bpy)_3_]^2+^. Importantly, it was also pointed out, as a helpful contrast to Oh and Boxer’s earlier work:^121^ “the EA spectrum revealing the shape of the second derivative of absorption band cannot be taken as direct evidence of the permanent dipole moment in the Franck–Condon excited state for a molecule with D_3_ symmetry, unless there is an asymmetric environment-induced distortion of the molecule.” Therefore, the present state of knowledge leans toward localization in [Ru­(bpy)_3_]^2+^ being solvent-induced rather than intrinsic. Notably, these findings are consistent with previous assertions of asymmetrical ligand-solvent interactions resulting in symmetry reduction, and hence, localization; details are compiled in an exhaustive earlier technical review by Yersin et al.^134^

It is remarkable that new fundamental insights continue to emerge after decades of research into the complex. A complete understanding of the nature of the excited state, particularly with respect to the charge distribution, of course, remains critical for the efficient design of solar-powered devices. Clarity about the subtle intricacies of the MLCT state notwithstanding, extensive research has taken place over the last several decades for its utilization. Indeed, the field of solar energy conversion, propelled initially by the oil crisis, oftentimes saw [Ru­(bpy)_3_]^2+^ at the epicenter due to its promising photophysical properties. We summarize cornerstone developments in the following sections.

## Photoelectrochemistry

Mechanistic investigations in the field of electrochemiluminescence (ECL) – which would serve as precursors to the study of sensitization of interfaces – started in the 1960s, focusing on organic compounds.^135–137^ However, reports of this phenomenon can be found in the literature already many decades earlier.^138–141^ In 1966, while researching new chemiluminescent systems, Hercules and Lytle had reported the first case of chemiluminescence from a TMC using [Ru­(bpy)_3_]^2+^.^142^ This paper later inspired Tokel and Bard to investigate the ECL properties of [Ru­(bpy)_3_]^2+^. In 1972,^143^ they reported the first case of ECL involving a TMC using [Ru­(bpy)_3_]^2+^. The paper also reports the first cyclic voltammogram of [Ru­(bpy)_3_]^2+^ (Figure 5a),^144^ followed in 1973 by a more detailed mechanistic investigation involving other chelate complexes of ruthenium.^145^ Another key study involving the electrochemistry of [Ru­(bpy)_3_]^2+^ was published in 1983 by Ohsawa, DeArmond, Hanck et al. By measuring the low temperature voltammogram of [Ru­(bpy)_3_]^2+^, they managed to observe six reversible reduction peaks corresponding to the double reduction of each bpy ligand, confirming the existence of isolated orbitals on each ligand, as suggested by the previous photophysical characterization (Figure 5b).^146^ In a more general context, this study was part of research on the redox properties of polypyridines. A field that has existed since the 1960s and that eventually led to the formulation of the concept of ligand-based redox series.^147^

**Figure 5:**
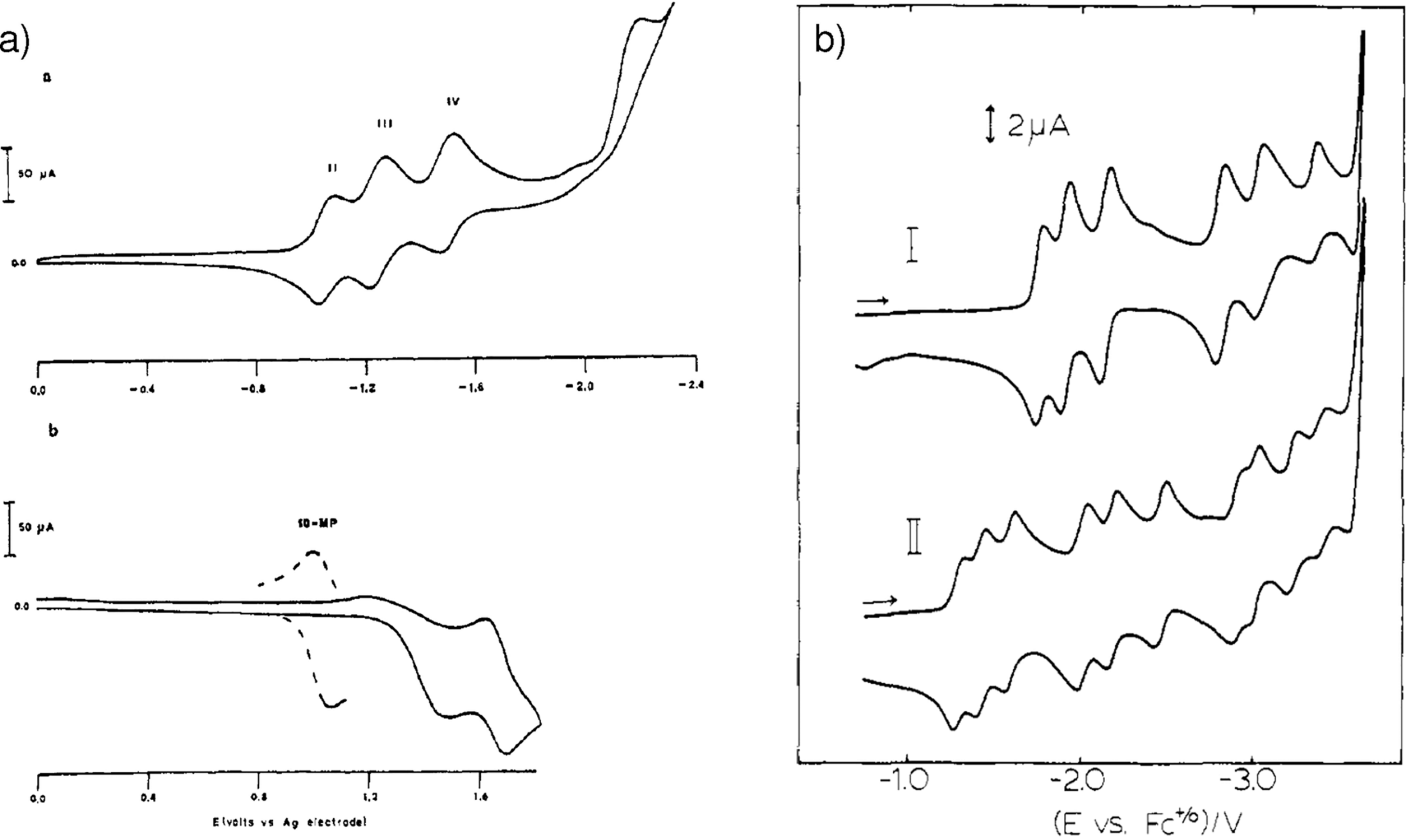
(a) First cyclic voltammogram of [Ru­(bpy)_3_]^2+^ reported by Tokel and Bard in 1972. Reproduced from ref 144. Copyright 1972 American Chemical Society. (b) Low temperature cyclic voltammogram of (I) [Ru­(bpy)_3_]^2+^ and (II) [Ru­(4,4′-(CO_2_Et)_2_bpy)_3_]^2+^ reported by Ohsawa, DeArmond, Hanck et al. in 1983. Reproduced from ref 146. Copyright 1983 American Chemical Society.

Since then, [Ru­(bpy)_3_]^2+^ has become the benchmark for the development of ECL systems^148,149^ and it has even found commercial applications in analytical instruments since the 1990s, thanks to its ECL properties.^150^ Commercial applications of ECL sensors based on [Ru­(bpy)_3_]^2+^ are still pursued today, with a recent publication reporting the detection of the SARS-CoV-2 Spike protein by a derivative of [Ru­(bpy)_3_]^2+^.^151^ Other avenues of implementation which exploit the unique electrochemical and photophysical properties of [Ru­(bpy)_3_]^2+^ include creation of molecular machines,^152,153^ simple logic-gates,^154^ and “artificial fireflies”.^155^

These more fundamental electrochemical studies would stimulate work on devices with the aforementioned backdrop of the oil crisis. In 1975, Gerischer proposed, based on a previous observation by Fujishima and Honda,^85^ a new model of solar cells based on a liquid junction.^156^ At the time, this device was purely built out of semiconductors without the addition of any dye. The same year, Gleria and Memming, inspired by the previous publications on the photo- and electrochemical properties of [Ru­(bpy)_3_]^2+^, reported the sensitization of SnO_2_ using [Ru­(bpy)_3_]^2+^.^157^ This paper represents the first step in the expansion of the study of photoinduced electron transfer at semiconductor interfaces since, until this point, such experiments were limited to organic dyes,^158–161^ with the only exception of chlorophyll and phthalocyanines for studies on photosynthesis.^162–165^ The following year, in 1976, Osa and Fujihira reported the first dye-sensitized solar cell based on rhodamine B immobilized on SnO_2_ and TiO_2_.^166^ The grafting of molecules on semiconductors was at the time investigated for the fabrication of more selective and efficient photoelectrochemical cells.^167^ The year after, Clark and Sutin reported the first experiment in which TiO_2_ was sensitized using [Ru­(bpy)_3_]^2+^ in solution phase.^168^ In this paper, they also highlighted the potential application for water splitting in the context of the research on artificial photosynthesis.

This study inspired further research in the field of photoelectrochemical cells for water splitting. In 1979, Hamnett, Goodenough et al. reported more detailed mechanistic studies on TiO_2_ sensitization by [Ru­(bpy)_3_]^2+^ in an attempt to improve photoelectrochemical production of H_2_.^169^ In this study, they acknowledge the importance of the study by Clark and Sutin, since up to that point, only organic dyes had been used for this application.

The studies reported to date always involved [Ru­(bpy)_3_]^2+^ as a free-floating species in solution. In order to improve the efficiency of photoelectrochemical cells, strategies to anchor [Ru­(bpy)_3_]^2+^ to the electrode interfaces started to be investigated. Shortly after the paper by Hamnett, Goodenough et al. in 1979, the first hybridization of Rubpy to a crystalline TiO_2_ surface was reported by the same group.^170^ A follow-up study showed ultrafast injection in the conduction band of the electrode.^171^ At the same time, Memming and Schröppel reported the sensitization of SnO_2_ with a monolayer formed by a surfactant derivative of [Ru­(bpy)_3_]^2+^.^172^ In both cases, only one of the bpy ligands was modified with the groups needed to form the monolayer. A significant improvement was introduced in 1985 by Desilvestro, Grätzel et al. They reported the sensitization of colloidal TiO_2_ with a derivative of [Ru­(bpy)_3_]^2+^ bearing two carboxylate groups on each bpy ligand – a change that would later prove instrumental for improving efficiencies.^173^ These investigations were still focused on the photoelectrochemical applications of [Ru­(bpy)_3_]^2+^.

The information gathered so far on semiconductor sensitization by [Ru­(bpy)_3_]^2+^ was then implemented in the field of photovoltaic devices. In 1988, Vlachopoulos, Grätzel et al. reported a DSSC based on the previously reported [Ru­(bpyCOOH)_3_]^2+^ linked to rough TiO_2_ that set the record efficiency for such devices at the time.^174^ It is important to notice that the authors of this paper highlight how the photophysical and redox properties of [Ru­(bpy)_3_]^2+^ are attractive for photovoltaic applications. In 1990, Nazeeruddin, Gratzel et al. reported a highly efficient DSSC which employed trinuclear Ru-complexes attached to TiO_2_.^175^ Photocurrent efficiencies were over 80%, and the best results were achieved with [RuL_2_(μ-(CN)­Ru­(CN)­L′)_2_], where L is 2,2′-bipyridine-4,4′-dicarboxylic acid and L′ is 2,2′-bipyridine. The fill factor was 75% and the power conversion efficiency was 11.2% at 520 nm. These numbers are comparable to the famed study of 1991 by O’Regan and Grätzel, which presented a watershed moment in the field,^176^ that has since expanded considerably,^177,178^ with Ru bipyridine dyes quickly becoming the benchmarking standard.^179,180^

## Photoredox Catalysis

At the end of the 1970s, Hedstrand, Kellog et al.^181^ and Van Bergen, Kellog et al.^182^ published what can be considered the first papers in the field of photoredox catalysis. They reported that the reduction of sulfonium ions by dihydropyridines could be enhanced upon light irradiation in the presence of catalytic amounts of [Ru­(bpy)_3_]^2+^, tetraphenylporphyrin, or eosin, with the former providing the largest rate enhancement. At the same time, DeLaive, Giannotti, and Whitten reported the photoredox reactivity of functionalized [Ru­(bpy)_3_]^2+^ with bulky substituents to disfavor back electron transfer.^183^ In their paper, they highlight how this new line of research was made possible by the previous studies on excited-state quenching via electron transfer. In a following paper, they also stated: “The attraction of [Ru­(bpy)_3_]^2+^, as well as analogues with osmium and iridium, is due to its strong absorption properties throughout the visible region, relatively long excited-state lifetime, and luminescence.”.^184^ In the 1980s, more papers started to appear in the literature about different types of oxidation and reduction reactions driven by the excited state of [Ru­(bpy)_3_]^2+^ (Figure 6).^185–187^ Again, at the end of the 2000s, when photoredox catalysis started to gain momentum in the scientific community, many studies were using [Ru­(bpy)_3_]^2+^ as a photocatalyst.^188–190^ This choice was also determined by the fact that [Ru­(bpy)_3_]^2+^ and other photosensitizers started to become commercially available around this time.^191^ For a more comprehensive review of the development of photoredox catalysis, the interested readers are referred to the review by Shaw et al.^192^

**Figure 6:**
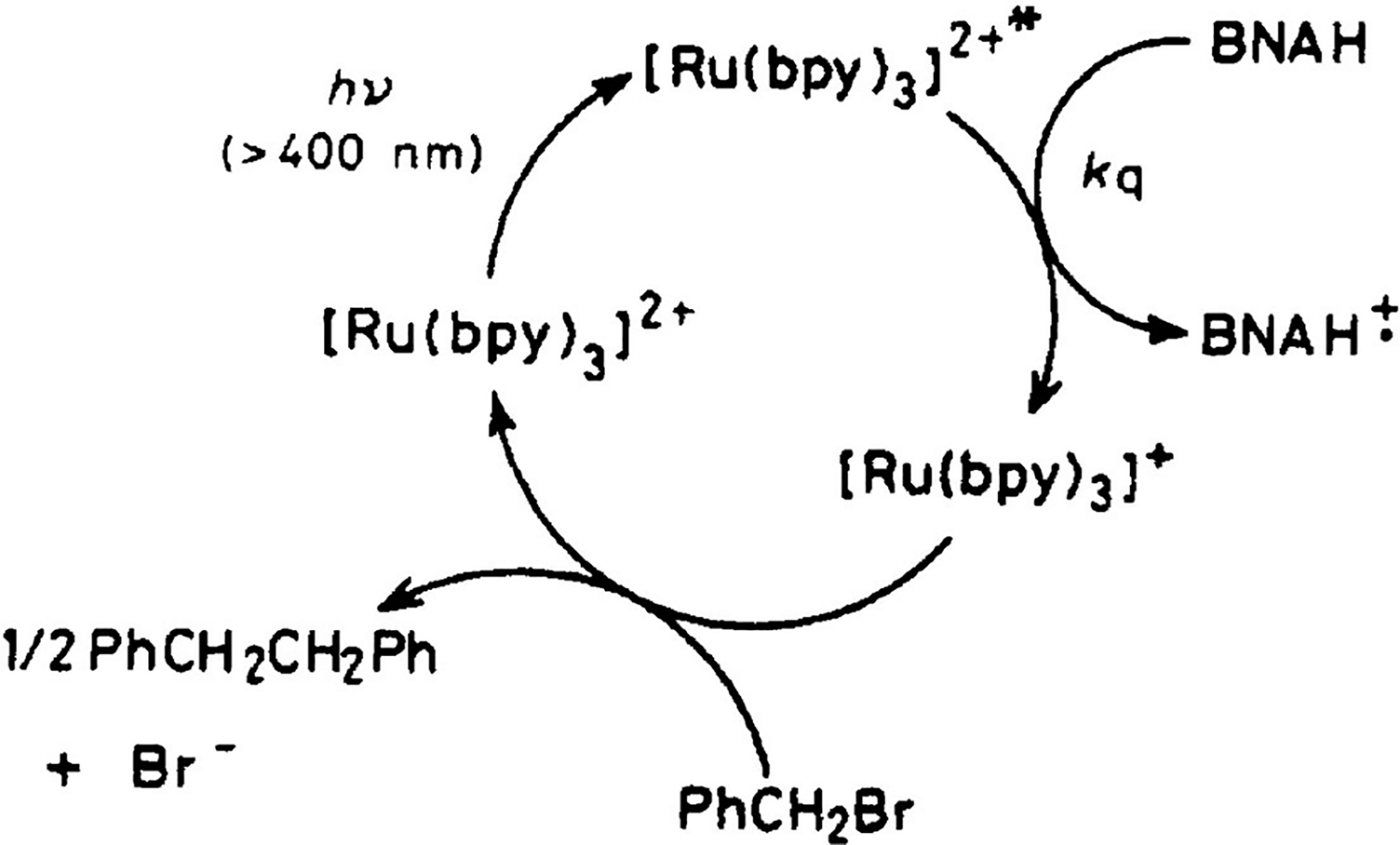
One of the earliest reported schemes for a photoredox reaction. In this process, [Ru­(bpy)_3_]^2+^ is first excited, and then it is reductively quenched by 1-benzyl-l,4-dihydronicotinamide (BNAH). Finally, [Ru­(bpy)_3_]^+^ reacts with benzyl bromide yielding 1,2-diphenylethane, the desired reaction product, and regenerating [Ru­(bpy)_3_]^2+^. Reproduced with permission from ref 186. Copyright 1984 Royal Society of Chemistry.

## Biochemical Studies

Complexation of transition metal ions was not only a property utilized in the context of fundamental analytical and inorganic chemistry, but could readily be implemented in a biological context. In 1938, a few years after a series of studies by Hammett and Walden reporting the oxidometric utility of [Fe­(bpy)_3_]^2+^, Beccari published the first of a series of papers about its biological activity.^193–196^ In 1951, Dwyer and co-workers - seemingly independently from Beccari^197^ – started studying how the chirality of metal complexes influences their interactions with chiral ions.^198^ This led him and others to study the effect of enantiopure complexes on mice, bacteria, and proteins, and among the complexes used in these papers, there was [Ru­(bpy)_3_]^2+^.^199^ This study was presumably inspired by the similarity that Dwyer saw between phenanthroline complexes and protonated strychnine – a similarity confirmed by a taste test on [Ni­(phen)_3_]^2+^!^200^

This study is now considered the start of the research on Ru-based drugs.^201–204^ This line of research was then continued by Dwyer’s collaborators after his death in 1952 with studies on the effect of TMCs (especially Ru and Fe-based ones) on several biological systems.^205–209^ Thanks to their previous studies on TMCs, discussed in the section on Analytical chemistry, the authors could conclude that the biological activity of TMCs was a consequence of both the charge on the metallic cation and the properties of the ligand, since their dissociation constants and tendency to racemize are negligible. These studies on metallodrugs were then overlooked until cis-platinum was later discovered in 1965, sparking renewed interest in the field of metallodrugs.^202,210,211^ As of October 2024, Web of Science returns more than 19,000 entries for “platinum drug*”, and only 4,800 for “ruthenium drug*”, highlighting the shift in focus. Nevertheless, Ru-polypyridines have remained complexes of choice for incursions into various fields, if not the first ones to be used. As such, while hematoporphyrins were the first photosensitizers used in photodynamic therapy,^212,213^ [Ru­(bpy)_3_]^2+^ and derivatives have also been investigated.^214–216^

Another subfield where [Ru­(bpy)_3_]^2+^ was at the heart of critical development, however, is the study of electron transfer in proteins. Marcus developed his theory of electron transfer in the 1950s.^217–219^ Subsequently, experimental research on the mechanism of electron transfer grew in popularity. At around the same time, in the 1960s, mechanistic studies on electron transfer in proteins started to appear in the literature.^220–223^ In 1977, Sutin observed quenching of [Ru­(bpy)_3_]^2+^ by cytochrome c. The interpretation of the study was, however, potentially affected by energy transfer.^224^ In 1982, English, Gray et al. reported the quenching via electron transfer of [Ru­(bpy)_3_]^2+^ by the copper blue protein, even though they could only measure diffusion-controlled rate constants.^225^ Prior to this, studies were performed using stopped-flow and TMCs as initiators.^226–228^

In 1982, Winkler, Gray et al. published a pioneering study in which [Ru­(bpy)_3_]^2+^ was quenched via electron transfer by ferricytochrome c bearing an artificial Ru cofactor located at a precise position in the protein structure.^229^ The combination of highly tunable artificial cofactors and [Ru­(bpy)_3_]^2+^ as a photoinitiator of electron transfer allowed Gray and co-workers to precisely control the protein systems,^230^ leading to significant insight in this field that was even recognized by Marcus in his Nobel lecture.^231^ By developing the flash-quench method, Chang, Gray and Winkler were able to further expand the range of potentials to study electron transfer.^232^ In these experiments, the Ru photosensitizer is first quenched so that the electron transfer with the redox cofactor of the protein involves either Ru­(III) or Ru­(I), achieving higher driving forces thanks to the stability of the oxidized and reduced versions of the Ru polypyridines. This technique allowed the determination of the rate of activationless electron transfer in several proteins (Figure 7a).^233^

These studies eventually led to a debate on the very fundamental nature of electron transfer in biology into the early 2000s. One prevailing view proposed that the protein scaffold enabled fine-tuning of parameters such as electronic coupling and reorganization energy for optimization of ET rates, as evidenced from site-specific mutagenesis studies.^230^ In contrast, an alternative hypothesis – largely advanced by Dutton and co-workers^234,235^ – instead argued for a simpler design principle of redox centers being positioned at distances <14 Å, with tunneling rates between them being robust to large variations in parameters such as packing fraction and free energy. Nowadays, using [Ru­(bpy)_3_]^2+^ and other photosensitizers has become a standard procedure to study electron transfer in artificial and natural proteins.^236,237^

**Figure 7:**
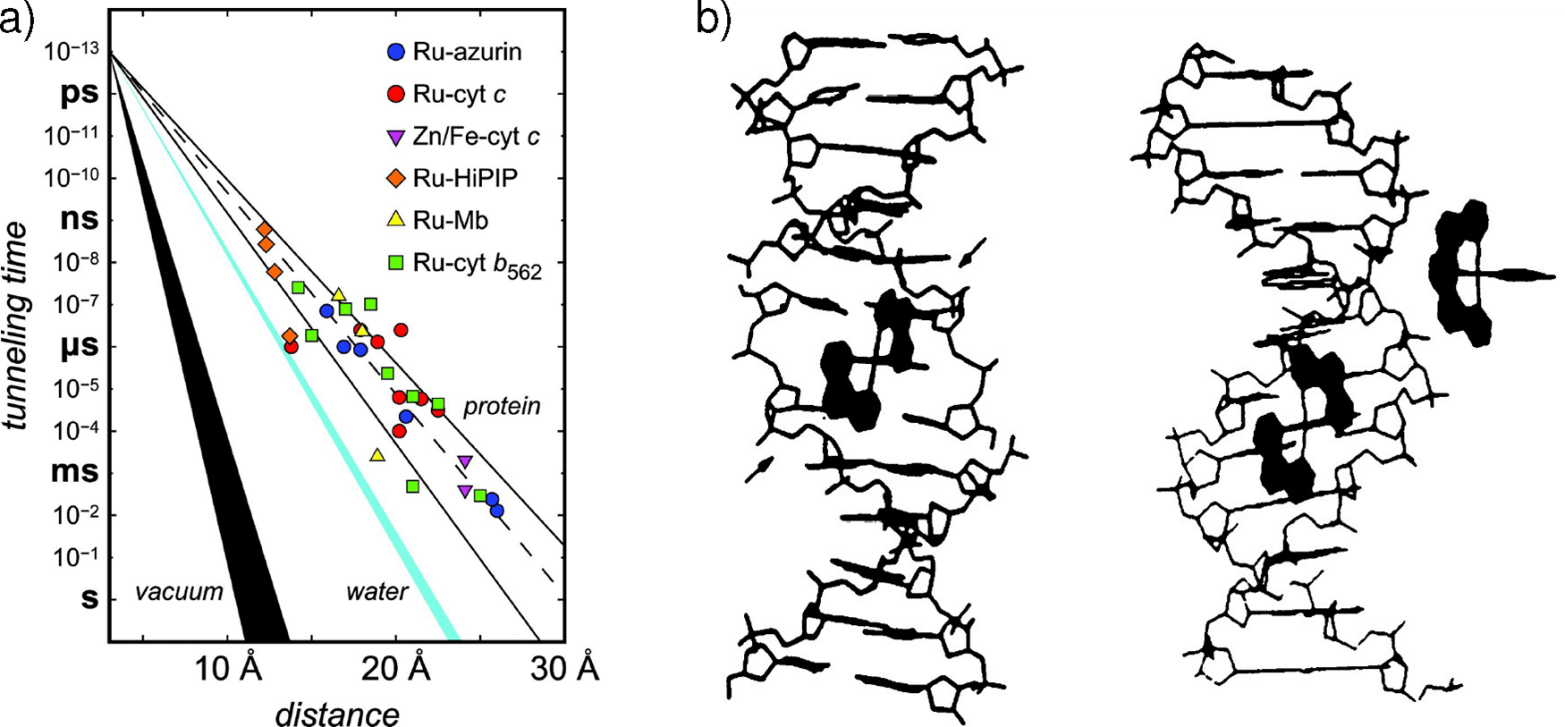
(a) Tunneling timetable for intraprotein electron transfer in Ru-modified proteins. Reproduced with permission from ref 233. Copyright 2005 National Academy of Sciences, U.S.A. (b) Model for the interaction of Δ-[Ru­(phen)_3_]^2+^ (left) and Λ-[Ru­(phen)_3_]^2+^ (right) with the DNA double helix. Reproduced with permission from ref 238. Copyright 1988 American Association for the Advancement of Science.

Ru complexes, in addition to their applications in the study of proteins, were also used to investigate other classes of biomolecules. In the quest to identify alternatives to radioactive probes to study DNA, scientists started to develop fluorescent probes based on the principle of intercalation between the DNA bases. As in the case of metallodrugs, the most explored class of intercalation probes were Pt complexes.^239^ However, Ru polypyridines were studied as well for this application. The first example of this kind was the interaction of [Ru­(phen)_3_]^2+^ with DNA reported by Barton et al. in 1984.^240^ As for many other cases reported in this study, the motivations behind the choice of Ru polypyridines for this application were “(i) the kinetically inert character of the low-spin d^6^ species, (ii) their intense metal to ligand charge-transfer (MLCT) band in the visible spectrum and since (iii) many chemical and spectroscopic properties of the poly­(pyridine) complexes have been established.”. Moreover, the lack of racemization of these compounds (reported by Dwyer, as mentioned at the beginning of this review) was also considered an attractive characteristic.^240^ In 1990, Friedman, Barton et al. also designed a new DNA intercalation complex based on [Ru­(bpy)_3_]^2+^ that has since then gained popularity in the field: [Ru­(bpy)_2_(dppz)]^2+^ (where dppz stands for dipyrido­[3,2-a:2′,3′-c]­phenazine).^241,242^

The studies on intercalation of Ru complexes in the DNA structure led to the use of such complexes for the investigation of the then-controversial field of long-range electron transfer through DNA.^243^ In 1986, Barton, Kumar, and Turro showed that DNA affects the electron transfer between Ru polypyridines, among which there was [Ru­(bpy)_3_]^2+^, and other inorganic electron acceptors.^244^ This study was followed by another paper in 1988, in which Purugganan, Turro, Barton et al. demonstrated that this effect was indeed due to DNA mediating the transfer of electrons.^238^ These pioneering papers have since then paved the way to a significant number of studies involving electron transfer involving DNA, ranging from biochemical studies^245^ to molecular electronics.^246^

Finally, [Ru­(bpy)_3_]^2+^ found its way also outside photochemistry laboratories, entering structural biology departments. In 1999, Fancy and Kodadek demonstrated for the first time the possibility of stabilizing protein structures via photoinduced cross-linking using [Ru­(bpy)_3_]^2+^.^247^ Since then, this method has become more commonly used among biochemists to investigate the structure of proteins and to stabilize metastable species, such as amyloid oligomers.^248^

## Conclusions

The preceding sections are a testament to the transformative impact of [Ru­(bpy)_3_]^2+^ on several branches of chemistry. Perhaps even more telling is the fact that the humble octahedral complex is the only one to have been inducted into popular culture. Indeed, it has a vibrant red cocktail named after it in Caltech’s Athenaeum,^249^ has found its way on number plates (Figure 8), and is said to “crystallize from thin air” in certain university campuses where it has been the focal point of research.^250^

**Figure 8:**
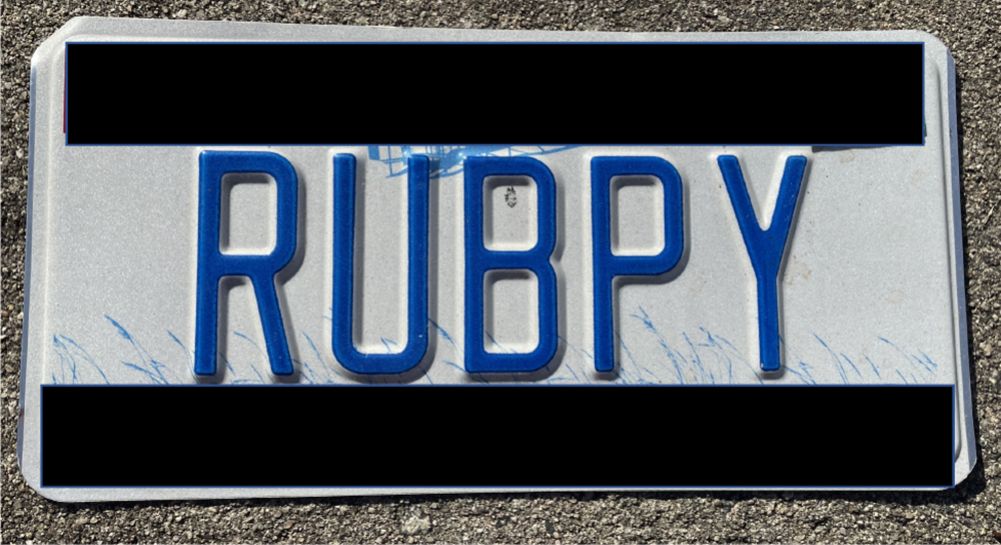
License plate of Prof. Thomas Meyer (picture kindly provided by Tyler Meyer).

Having lived many lives, some of which are ongoing, the extensive research on [Ru­(bpy)_3_]^2+^ has elevated it to the status of a reference standard, the closest one might get to scientific immortalization. Not only is it a benchmark for new earth-abundant sensitizers being presently discovered,^251–253^ it has also been reported as standard for the determination of emission quantum yields,^254^ cage escape yields,^255^ as well as for differential extinction coefficients in transient absorption spectroscopy,^256^ making it an actinometer of choice. It has also been used as a standard template for mechanistic investigations in photoredox catalysis.^257^

As noted previously, there is a renewed scientific interest in [Ru­(bpy)_3_]^2+^’s own fundamental photophysics in recent years, particularly with respect to the nature of the localization of the charge-transfer state on early time scales. The lively debate has continued for several decades. Insights, even if not outright conclusive, have been instrumental for understanding TMC photophysics generally. We are confident that the very detailed characterization information already available on [Ru­(bpy)_3_]^2+^ will encourage its investigation using even more advanced spectroscopic techniques: some studies on its derivatives having already been carried out.^258–263^ Ultimately, these future studies should stand to resolve not only its own fundamental photophysics but also establish it as a standard template for tackling new, unknown systems. Yet another life for [Ru­(bpy)_3_]^2+^, we conclude, seems inevitable to live.
